# Hip Fractures and Bone Mineral Density in the Elderly—Importance of Serum 25-Hydroxyvitamin D

**DOI:** 10.1371/journal.pone.0091122

**Published:** 2014-03-12

**Authors:** Laufey Steingrimsdottir, Thorhallur I. Halldorsson, Kristin Siggeirsdottir, Mary Frances Cotch, Berglind O. Einarsdottir, Gudny Eiriksdottir, Sigurdur Sigurdsson, Lenore J. Launer, Tamara B. Harris, Vilmundur Gudnason, Gunnar Sigurdsson

**Affiliations:** 1 Unit for Nutrition Research, University of Iceland and Landspitali University Hospital, Reykjavik, Iceland; 2 Icelandic Heart Association Research Institute, Kopavogur, Iceland; 3 Intramural Research Program, Laboratory of Epidemiology, Demography and Biometry, National Institute of Aging, Bethesda, Maryland, United States of America; 4 Division of Epidemiology and Clinical Applications, National Eye Institute, Bethesda, Maryland, United States of America; 5 University of Iceland, Reykjavik, Iceland; Baylor College of Medicine, United States of America

## Abstract

**Background:**

The significance of serum 25-hydroxyvitamin D [25(OH)D] concentrations for hip fracture risk of the elderly is still uncertain. Difficulties reaching both frail and healthy elderly people in randomized controlled trials or large cohort studies may in part explain discordant findings. We determined hazard ratios for hip fractures of elderly men and women related to serum 25(OH)D, including both the frail and the healthy segment of the elderly population.

**Methods:**

The AGES-Reykjavik Study is a prospective study of 5764 men and women, age 66–96 years, based on a representative sample of the population of Reykjavik, Iceland. Participation was 71.8%. Hazard ratios of incident hip fractures and baseline bone mineral density were determined according to serum concentrations of 25(OH)D at baseline.

**Results:**

Mean follow-up was 5.4 years. Compared with referent values (50–75 nmol/L), hazard ratios for hip fractures were 2.24 (95% CI 1.63, 3.09) for serum 25(OH)D <30 nmol/L, adjusting for age, sex, body mass index, height, smoking, alcohol intake and season, and 2.08 (95% CI 1.51, 2.87), adjusting additionally for physical activity. No difference in risk was associated with 30–50 nmol/L or ≥75 nmol/L in either model compared with referent. Analyzing the sexes separately, hazard ratios were 2.61 (95% CI 1.47, 4.64) in men and 1.93 (95% CI 1.31, 2.84) in women. Values <30 nmol/L were associated with significantly lower bone mineral density of femoral neck compared with referent, z-scores -0.14 (95% CI −0.27, −0.00) in men and −0.11 (95% CI −0.22, −0.01) in women.

**Conclusions:**

Our results lend support to the overarching importance of maintaining serum 25(OH)D above 30 nmol/L for bone health of elderly people while potential benefits of having much higher levels could not be detected.

## Introduction

The importance of vitamin D for skeletal health has long been recognized, and serum 25-hydroxyvitamin D [25(OH)D] is the generally accepted indicator of vitamin D status. Still, optimal serum concentration for bone health is a subject of active debate and intensive research [1]. The Institute of Medicine recently recommended serum levels of 40–50 nmol/L for most adults, based on multiple skeletal outcomes [2], while other experts have suggested levels as high as 75 to 100 nmol/L [3], [4].

Several functional indicators are used for vitamin D adequacy as it relates to bone health of the elderly, including bone mineral density and prevention of bone loss [5], [6], as well as surrogate measures such as maximal calcium absorption and suppression of parathyroid hormone levels [4], [7]. However, fracture prevention, and the levels of 25(OH)D associated with decreased fracture risk may be a more relevant and clinically useful functional marker, as it relates directly to health and quality of life of the elderly. Still, associations between circulating 25(OH)D concentrations and incident fractures in old age remain uncertain [5], [6], [8]. While several prospective cohort studies have reported higher risk for fractures at lower 25(OH)D concentrations [3], [9]–[14], others show no such association [15]–[17]. Further, the reported thresholds of significance for fracture risk are quite varied, ranging from 30–40 nmol/L (11) to a high of 90–100 nmol/L [3].

Hip fracture risk of the elderly is a function of multiple factors, including bone mineral density, muscle strength and balance, all of which have been related to vitamin D status and function [9], [18]. In nine out of ten instances, hip fracture is sustained through a fall [19], and risk of falling has been related to vitamin D status [20].

Adding to this uncertainty, Randomized controlled trials (RCTs), often considered to provide the highest quality evidence, have not shown a consistent benefit from vitamin D supplements for hip bone mineral density (BMD) or fracture prevention, except possibly in the institutionalized elderly [21] or when given in combination with calcium [22]. Conversely, a recent meta-analysis of RCTs, estimating actual intake of vitamin D rather than prescribed dose, concluded that intakes ≥800 IU/day may be somewhat favorable in the prevention of hip fractures and other non-vertebral fractures in adults 65 years or older [23].

Given the social and economic burden of hip fractures in the elderly, prevention and risk reduction are of prime importance. Sufficient doses of vitamin D for skeletal health in old age need particular elucidation. However, high-risk groups, such as the frail elderly, may be difficult to reach in large cohort studies or RCTs of community-living adults, which in turn may have contributed to the discordant findings of previous studies.

Here we report on the risk of incident hip fractures in the elderly related to circulating 25(OH)D concentrations and bone mineral density in participants in the large prospective AGES-Reykjavik Study, selected at random from the general population.

## Methods

### Participants

The Ages Gene/Environment Susceptibility (AGES) -Reykjavik Study [24] is a follow-up of the population based Reykjavik Cohort Study, initiated in 1967.

A total of 27,281 men and women, all Caucasians, born in 1907–1935 and residing in Reykjavik and nearby communities were selected at random from the national registry. From these, 19,381 attended the study during the period 1967 to 1991 [25]. From the 11,549 cohort members still alive when AGES-Reykjavik Study examinations began in September 2002, 8,030 individuals were randomly chosen and invited to the study. From these, 5,764 participants, age 66–96 years (mean age 76 years), had enrolled in the AGES-Reykjavik Study by January 2006 (71.8% of invited), with continuous recruitment throughout the period. Participants were offered free taxi rides to the clinic at every visit and personal assistance from the drivers in order to better reach those with limited mobility.

### Ethics statement

Written informed consent was obtained from all participants, and the study was approved by the Icelandic National Bioethics Committee (VSN: 00-063) and the National Institute on Aging Intramural Institutional Review Board (MedStar IRB for the Intramural Research Program, Baltimore, MD).

### Clinical data collection

Extensive data were collected in the AGES-Reykjavik Study during a series of clinical examinations, according to standardized study protocols by staff of the Icelandic Health Association. Data were gathered on smoking, alcohol intake and consumption frequency of selected foods, including milk and dairy products, using a validated food frequency questionnaire [26]. Physical activity was assessed by self-reported level of physical activity during the last 12 months, where participants were asked how frequently in times per month, week or day they engaged in moderate or vigorous activity, giving examples of this level of activity for clarification. Participants got personal assistance in answering questions. Quantitative computed tomography (QCT) -scanning was performed for BMD measurements.

Of the 5,764 participants of AGES-Reykjavik, 303 did not meet the inclusion criteria, e.g., lacking serum 25(OH)D measurements, leaving 5461 individuals included in the study, all with available fracture data. Of these, 679 individuals did not undergo QCT scanning, leaving 4782 individuals for the analysis of BMD and vitamin D status.

### Measurement of serum 25(OH)D

Blood was collected during the first clinic visit to AGES-study, from September 2002 to January 2006, and fasting serum samples were kept frozen at −80°C on-site in the IHA laboratory. Quantitative determination of total 25(OH) D (D_2_ and D_3_) was conducted by means of a direct, competitive chemiluminescence immunoassay (CLIA), using the LIAISON 25 OH Vitamin D Total assay (DiaSorin, Inc., Stillwater, Minnesota). The inter assay coefficient of variation was <6.5%, using a previously frozen serum pool as the control sample and <12.7% when the calculated data were from measurements using Liaison quality controls.

### Bone mineral density measurements

During the first clinic visit, QCT measurements, providing true volumetric density, were taken on the left hip, using a 4-detector CT system (Sensation, Siemens Medical Systems, Erlangen, Germany). Using a standardized protocol, scans were obtained encompassing the proximal femur from a level 1 cm above the acetabulum to a level 5 mm inferior to the lesser trochanter with 1 mm slice thickness. To calibrate CT Hounsfield units to equivalent bone mineral concentration, all subjects were scanned with a calibration phantom (Image Analysis, Columbia, KY, USA). Further procedures and quality assessments have been described in detail elsewhere [24], [27].

The variables used in the present study are volumetric integral BMD (mg/cm^3^), reflecting both trabecular and cortical bone mass, of the femoral neck and trochanteric region separately. Reasons for exclusion from the QCT were inability to lie supine, body weight over 150 kg or having undergone hip replacement surgery.

### Fractures

Hip fracture data were recorded, verified and confirmed from medical and radiological records as previously described [28]. Incident fractures were recorded from participants' enrollment into the study until 31^st^ of December 2009, with a mean follow-up time of 5.4 years (SD 1.5). Hip fractures were defined according to International Classification of Diseases version 10, diagnostic codes S72.0, S72.1, S72.2 (WHO 1994). The AGES-Reykjavik Study fracture registration has been shown to have a capture rate of about 97% for hip fracture [28].

### Statistical analyses

The mean and standard deviation (SD) were used to describe continuous variables and percentages were used for dichotomous variables. Serum 25(OH)D was categorized, *a priori*, into four groups of <30, ≥30–50, ≥50–75 and ≥75 nmol/L. The group ≥50–75 nmol/L (sufficient level) was used as referent, to establish whether beneficial effects could be detected at higher levels (≥75 nmol/L) and to compare with lower levels, (30–50 nmol/L) and (<30 nmol/L), concentrations that have been considered inadequate and depleted, according to the Institute of Medicine [2]. The absolute BMD values (in mg/cm^3^) of the femoral neck and trochanteric area were transformed into a gender-specific z-score, based on the internal distribution in our study population. The z-score for femoral neck was used as the primary outcome measure when examining the association between 25(OH)D and BMD, which was performed as a secondary analysis.

Cox Proportional Hazard Model was used when examining the association between 25(OH)D and bone fractures; and linear regression when examining the association with bone mineral density. As a measure of association we used χ^2^-test (Type III) for dichotomous outcomes and t-test for continuous outcomes under the null hypothesis that all 25(OH)D groups were equal (p for effect). For testing linear trend, ordinal values (0,1,2,3) for serum 25(OH)D were entered in the regression model. All of the analyses were carried out using SAS statistical software (version 9.2, SAS Institute, Cary, NC).

In our analyses we selected *a priori* and included the following set of covariates: age (continuous, no missing), sex (binary, no missing) height (continuous, 0.1% missing), current smoking (binary, 2.5% missing), body mass index (BMI) (<18.5, 18.5–24.9, 25–29.9, 30–34.9, 35–39.9 and 40+, 0.1% missing), current alcohol intake (0, 1–25, 26–50 and 50+ g/day, 3% missing), season (winter: Dec, Jan, Feb, spring: Mar, Apr, May, summer: June, July, Aug, autumn: Sept Oct Nov, no missing), current physical activity (never, rarely, occasionally, moderate, high, 4.0% missing). As self reported milk and dairy product intake and serum 25(OH)D at baseline were essentially uncorrelated (Spearman r = 0.06), milk and dairy product intake was not included as covariate. The proportion of subjects missing data on one or more covariate was 4.4%. Due to the relatively low number of missing values for most covariates, missing values were replaced with median (continuous) and most frequent (dichotomous) values. Two models were used for covariate adjustment: Model A adjusting for age, sex, smoking, BMI, height, alcohol intake and season of blood sample collection; and Model B, additionally adjusting for physical activity.

To check the robustness of our findings with respect to imputation for missing covariates and length of follow-up we 1) ran all analyses using complete case analyses (all subjects with missing covariates excluded); and 2) examined the association between baseline 25(OH)D with hip fractures, excluding all fractures occurring within 6 months from baseline.

## Results

A total of 261 hip fractures were encountered during a mean follow-up time of 5.4 years (SD 1.5). Characteristics of the study participants by sex and serum 25(OH)D are shown in **Table 1**. Fourteen per cent of men and 19% of women had serum 25(OH)D concentrations below 30 nmol/L, and 21% and 14% had concentrations ≥75 nmol/L, men and women respectively. There were significant negative trends associated with 25(OH)D category for the following variables in both men and women: BMI, % physically inactive, % drinking no alcohol and % smoking, while positive trends were found for BMD. There was no significant association between level of serum 25(OH)D and mean age for men or women.

**Table 1:** Characteristics of male and female participants at baseline according to categories of serum 25-hydroxyvitamin D (N = 5461), mean and SD.

	*Serum 25-hydroxyvitamin D (nmol/L)*				
	<30	30-<50	50-<75	≥75	P value^1^
**Males (n = 2346)**					
n (%)	337 (14%)	638 (28%)	886 (38%)	485 (21%)	
Femoral neck BMD, mg/cm^3^	245 (59)	253 (51)	254 (50)	259 (53)	0.0005
Trochanter BMD, mg/cm^3^	242 (51)	248 (51)	252 (47)	255 (51)	0.0002
Age in years	76.7 (5.8)	76.8 (5.5)	76.7 (5.4)	76.8 (5.4)	0.65
Height, cm	175.1 (6.2)	175.1 (6.2)	175.5 (6.2)	175.8 (6.2)	0.03
BMI, kg/m^2^	27.2 (4.3)	27.3 (3.9)	26.7 (3.7)	26.3 (3.5)	<0.0001
% Physically inactive	61	46	37	34	<0.0001
% Not drinking alcohol	34	30	24	27	0.002
% Current smokers	23	12	8	9	<0.0001
					
**Females (n = 3125)**					
n (%)	601 (19%)	992 (32%)	1103 (35%)	429 (14%)	
BMD for femoral neck (mg/cm^3^)	240 (50)	245 (52)	245 (49)	248 (48)	0.03
BMD for trochanter (mg/cm^3^)	221 (49)	227 (50)	227 (46)	229 (45)	0.02
Age in years	76.8 (5.6)	76.7 (5.8)	76.3 (5.7)	76.7 (5.6)	0.20
Height, cm	160.3 (5.7)	160.7 (5.6)	161.1 (5.9)	160.8 (6.1)	0.006
BMI, kg/m^2^	28.1 (5.5)	27.8 (4.9)	26.6 (4.4)	26.2 (4.3)	<0.0001
% Physically inactive	66	50	45	42	<0.0001
% Not drinking alcohol	54	41	35	37	<0.0001
% Current smokers	18	12	11	10	0.0001
					

1 T-test for trend for continuous covariates with serum 25-hydroxyvitamin D entered as ordinal values.

Chi-square test for dichotomous covariates.

For males the mean (SD) integral BMD was 254 (51)mg/cm^3^ for femoral neck and 250 (50)mg/cm^3^ for trochanter. The corresponding values for females were 244 (50)mg/cm^3^ and 226 (48)mg/cm^3^. The mean (SD) serum 25(OH)D was 57 (25)nmol/L and 51 (24)nmol/L for males and females, respectively.

**Table 2** shows hip fracture hazard ratios according to serum 25(OH)D categories for all participants and for men and women separately. Compared with referent values of 50–75 nmol/L, the risk for hip fractures more than doubled in all subjects with levels below 30 nmol/L, values remaining relatively stable through adjustment models. Adjusting for age, height and BMI, current smoking, season of blood sampling and alcohol intake, the hazard ratio was 2.24 (95% CI 1.63, 3.09) and with further adjustment for physical activity it was 2.08 (95% CI 1.51, 2.87). No increase in risk was associated with serum concentrations 30–50 nmol/L compared with referent values and neither was there any difference in risk associated with values ≥75 nmol/L in either adjustment model. When the sexes were analyzed separately, hazard ratio for men was 2.61 (95% CI 1.47, 4.64), compared with 1.93 (95% CI 1.31, 2.84) for women in the fully adjusted model. Again risk ratios did not change significantly between the different adjustment models in either sex. Values between 30–50 nmol/L and values ≥75 nmol/L were not associated with changes in risk compared with referent values in either sex.

**Table 2:** Hazard ratios for hip fractures according to serum 25-hydroxyvitamin D categories among all subjects (N = 5461), and stratified by sex.

		Unadjusted	Model A^2^	Model B^3^
		HR (95% CI)	HR (95% CI)	HR (95% CI)
*All Subjects*	cases/n (%)			
<30 nmol/L	77/938 (8.2%)	2.14 (1.57, 2.94)	2.24 (1.63, 3.09)	2.08 (1.51, 2.87)
30–50 nmol/L	71/1620 (4.4%)	1.10 (0.80, 1.52)	1.13 (0.82, 1.56)	1.11 (0.80, 1.53)
50–75 nmol/L	80/1989 (4.0%)	1.00	1.00	1.00
≥75 nmol/L	33/914 (3.6%)	0.91 (0.61, 1.37)	0.90 (0.60, 1.35)	0.94 (0.62, 1.41)
P for effect^1^		<0.0001	<0.0001	<0.0001
*Males*	cases/n (%)			
<30 nmol/L	25/337 (7.4%)	2.61 (1.49, 4.58)	2.81 (1.59, 4.96)	2.61 (1.47, 4.64)
30–50 nmol/L	24/604 (3.8%)	1.35 (0.77, 2.36)	1.52 (0.86, 2,67)	1.49 (0.84, 2.63)
50–75 nmol/L	25/886 (2.8%)	1.00	1.00	1.00
≥75 nmol/L	14/485 (2.9%)	0.99 (0.51, 1.90)	0.97 (0.50, 1.88)	1.02 (0.53, 1.97)
P for effect^1^		0.003	0.001	0.006
*Females*	cases/n (%)			
<30 nmol/L	52/601 (8.7%)	1.89 (1.29, 2.76)	2.06 (1.40, 3.02)	1.93 (1.31, 2.84)
30–50 nmol/L	47/992 (4.7%)	0.97 (0.66, 1.44)	1.01 (0.68, 1.50)	0.99 (0.67, 1.47)
50–75 nmol/L	55/1103 (5.0%)	1.00	1.00	1.00
≥75 nmol/L	19/429 (4.4%)	0.94 (0.56, 1.58)	0.86 (0.51, 1.46)	0.89 (0.53, 1.51)
P for effect^1^		0.001	0.0002	0.001

Abbreviations: HR Hazard Ratio, CI confidence interval.

1 Chi-square test (type 3).

2 Adjusted for age at recruitment, sex (when all subjects are included), height, body mass index, current smoking, season of blood sampling and alcohol intake.

3 Additionally adjusted for current physical activity.

Based on the prevalence of hip fractures in the 50–75 nmol/group for all subjects (4.0%) we estimated the excess number of fractures, i.e. expected minus observed fractures, in the <30 nmol/L group. Expected fractures were 38, or 0.04*938 (number of subjects with <30 nmol/L), while 77 fractures were observed. This suggests that 15% (or 39 excess fractures out of 261 in total) may be attributable to poor (<30 nmol/L) serum 25(OH) D status.

Associations between 25(OH)D concentrations and BMD at baseline in femoral neck are shown in **Table 3**, with BMD reported as z-scores. Using the values 50–75 nmol/L as referent, values below 30 nmol/L were consistently associated with slightly lower BMD z-score. In Model A, adjusting for age, height, BMI, smoking, alcohol intake and season, the adjusted difference in z-score was −0.18 (95% CI −0.31, −0.04) in men and −0.13 (95% CI −0.23, −0.03) in women. In model B, additionally adjusting for physical activity, the corresponding values were −0.14 (95% CI −0.27, −0.00) and −0.11 (95% CI −0.22, −0.01), men and women, respectively. No significant difference in risk was associated with other categories of 25(OH)D concentrations, except for a small increase at concentrations ≥75 nmol/L. Taking both sexes together, there was an increase of 0.10 (95% CI 0.02, 0.18) in Model A, with values remaining stable through further adjustment. When sexes were analyzed separately, only BMD z-scores for males remained significantly higher in the ≥75 nmol/L group than in the referent. Comparable values were found for BMD in trochanter associated with 25(OH)D concentrations in both sexes (data not shown). A total of 679 participants in the study did not undergo QCT scanning. Associations between hip fractures and 25(OH)D concentrations in the 4782 individuals with available scanning data were found to be similar to those observed in the larger group of 5461 participants.

**Table 3:** Differences in z-score (number of SD from age corrected mean) of femoral neck bone mineral density according to serum 25-hydroxyvitamin D categories at baseline (N = 4782).

	Unadjusted	Model A^2^	Model B^3^
	Δ(95% CI)	Δ (95% CI)	Δ (95% CI)
***Femoral neck BMD(z-score)***			
*All (N = 4782)*			
<30 nmol/L	−0.13 (−0.21, −0.04)	−0.15 (−0.23, −0.07)	−0.13 (−0.21, −0.05)
30–50 nmol/L	−0.02 (−0.09, 0.05)	−0.04 (−0.11, 0.03)	−0.03 (−0.10, 0.03)
50–75 nmol/L	Referent	Referent	Referent
≥75 nmol/L	0.08 (−0.01, 0.16)	0.10 (0.02, 0.18)	0.09 (0.01, 0.17)
P for effect^1^	0.0006	<0.0001	0.0001
*Males (n = 2104)*			
<30 nmol/L	−0.18 (−0.31, −0.04)	−0.18 (−0.31, −0.04)	−0.14 (−0.27, −0.00)
30–50 nmol/L	−0.02 (−0.13, 0.08)	−0.04 (−0.15, 0.07)	−0.03 (−0.13, 0.08)
50–75 nmol/L	Referent	Referent	Referent
≥75 nmol/L	0.10 (−0.02, 0.21)	0.12 (0.01, 0.24)	0.11 (−0.01, 0.22)
P for effect^1^	0.004	0.0009	0.01
*Females (n = 2678)*			
<30 nmol/L	−0.10 (−0.21, 0.01)	−0.13 (−0.23, −0.03)	−0.11 (−0.22, −0.01)
30–50 nmol/L	−0.01 (−0.11, 0.08)	−0.04 (−0.12, 0.05)	−0.03 (−0.12, 0.05)
50–75 nmol/L	Referent	Referent	Referent
≥75 nmol/L	0.06 (−0.06, 0.18)	0.07 (−0.04, 0.19)	0.07 (−0.04, 0.18)
P for effect^1^	0.14	0.01	0.03

Abbreviations: CI confidence interval.

1 F-test (type 3).

2 Adjusted for age at recruitment, sex (when all subjects are included), height, body mass index, current smoking, season of blood sampling and alcohol intake.

3 Additionally adjusted for current physical activity.

**Figure 1:**
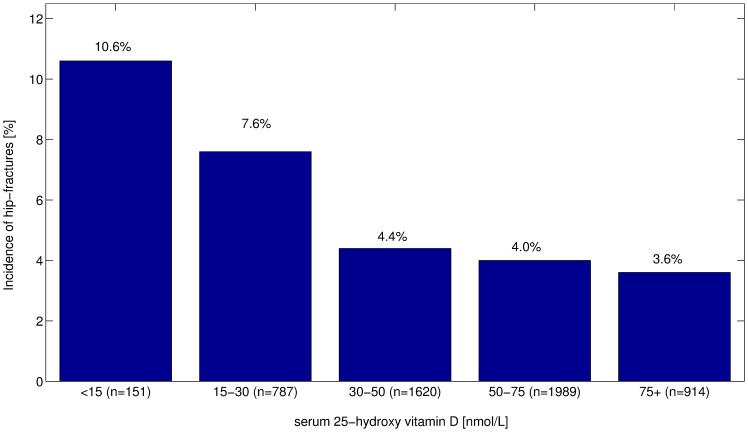
Hip fracture incidence (%) during a follow-up of 5.4 years (SD1.5) among AGES participants (N = 5461) according to serum baseline categories of 25(OH)D. Unadjusted values.

**Figure 1** shows the percentage of incident hip fractures according to baseline values of 25(OH)D in all participants, during a mean follow-up time of 5.4 years, mean time to event 3.4 years (SD1.8). A total of 10.6% of those with values below 15 nmol/L experienced a fracture, while corresponding proportions were 7.6% and 4.4% for those with values from 15–30 nmol/L and 30–50 nmol/L, respectively. There was non-significant change in the percentage at higher 25(OH)D values. However, mean time to fracture was not significantly different between the 25(OH)D groups, 3.1 years (SD 1.8) in the lowest category, compared with 3.9 years (SD 3.9), 3.3 years (SD 1.3) and 3.5years (SD 1.6) in the higher categories.

Finally we found little evidence to suggest that imputing values for missing covariates may have contributed to residual confounding. As an example, when comparing the referent with <30 nmol/L for both sexes using model B (Table 2) the hazard ratio for hip fracture was 2.08 (1.51, 2.87) and was essentially unchanged 2.08 (1.49, 2.91) when subjects with missing covariate information were excluded. Correspondingly when all individuals with hip fracture events occurring within 6 months (n = 14) were excluded (using imputed covariate values), the hazard ratio was 2.10 (1.51, 2.93). There was no trend with respect to time to fracture across categories of 25(OH)D. As an example the mean time to event was 3.1 years (SD 1.8) in the lowest (<30 nmol/L) compared with 3.3 years (SD 1.3) in the referent category (50 -<75 nmol/L).

## Discussion

In this prospective cohort study of 5461 elderly people experiencing a sizable number of hip fractures (n = 261), the estimated risk of hip fracture was twice as high in individuals with serum 25(OH)D concentrations below 30 nmol/L, using 50–75 nmol/L as a referent and adjusting for a series of relevant cofactors. The risk increase associated with low 25(OH)D levels was somewhat higher for men than women, but remained stable and highly significant in both sexes through various adjustments. No statistically significant difference in risk was associated with other categories of 25(OH)D concentrations, for levels either between 30 and 50 nmol/L or above 75 nmol/L, compared with the referent values.

The relevance of vitamin D status for hip fracture risk has been considered uncertain in large systematic reviews of vitamin D and bone health [5], [6]. Indeed, several cohort studies have found no associations between hip fracture risk and vitamin D [15]–[17], while some studies have reported risk protection by quite high serum 25(OH)D concentrations, up to 100 nmol/L [3], [10]. Still others show tendencies for increased risk at low levels below 30–40 nmol/L, with no further protection at higher concentrations [9], [11], [13]. According to a large multi-center study from Norway [14] there is a moderate increase in risk for hip fractures among those in the lowest quartile for baseline 25(OH)D (≤42.1 nmol/L) compared with the highest ≥67.9 nmol/L), with a hazard ratio of 1.34. Also, RCTs are not unequivocal in their conclusions, regarding either the efficacy or ideal dose of vitamin D supplements for fracture prevention [21], [22], [29]. Elucidating the significance of vitamin D for hip fracture risk of the elderly is of great public health significance, considering the public health costs, high mortality and human suffering related to hip fractures [30]. As vitamin D of varying doses is widely used in clinical practice to lower the risk of osteoporotic fractures, it is of prime importance to determine whether relatively high 25(OH)D concentrations are needed for risk protection, concentrations which generally require larger doses of vitamin D supplementation than the 800 IU currently recommended for the elderly by the Institute of Medicine [2] and recently endorsed in a review of randomized controlled trials [31]. Even though these higher doses are not considered toxic, adverse effects, such as increased risk of kidney stones, have been indicated in studies like the Women's Health Initiative [32]. The study of Sanders et al. [33] further alerts to possible adverse outcomes associated with large doses, as they reported increased risk for falls and fractures in a group of elderly people receiving 500,000 IU of vitamin D annually for 3 to 5 years.

Differences in previous study outcomes have been explained in part by the different populations under study as well as possible differences in confounder adjustments [5]. Specifically, the frail and sickly may not be included or reached in all community-based studies of the elderly, but lower vitamin D status is consistently reported among the frail compared with those more physically active and in better health [3], [9]. Thus, the segment of the elderly population having the lowest 25(OH)D levels may not be included in many cohort studies and the significance of very low values thus overlooked or minimized [9], [11]. In our study, which was based on a random sample from the general population, the participation rate was high, or 71.8%, and the distribution of 25(OH)D concentration was quite wide, with comparable proportions of the population having values below 30 nmol/L and above 75 nmol/L. A significant proportion of all hip fractures in our study (15%), may thus be attributed to poor or deficient (<30 nmol/L) serum 25(OH)D. Interestingly, this is a comparable proportion to that reported from combined calcium and vitamin D supplementation for reducing fracture risk [34]. Possibly the relatively high calcium intake in our population, due to widespread use of milk and dairy products, may contribute to this effect [7].

The fracture incidence observed in our study, where both sexes were taken together for improved statistical power, also strongly indicates that there is a nonlinear relationship between circulating 25(OH)D and fracture risk, with only small gains being associated with serum levels above 50 nmol/L. The highest incidence was observed in the group with 25(OH)D values below 15 nmols, with steeply decreasing proportion with increased circulating vitamin D up to 50 nmol/L, but little differences above those concentrations.

While appropriate adjustments were made for season in our analysis, seasonal variation in 25(OH)D values was less pronounced in the present study than previously reported for younger populations in Reykjavik, geographically located at 64° N [7]. The difference in mean values obtained in summer and winter was only 4 nmol/L in this elderly population. The relative lack of seasonal variation indicates that vitamin D intake may play a more important role for vitamin D status in this population compared with younger people. Indeed, cod liver oil, either as liquid or capsules, is a common supplement in Iceland, with 61% of this population reporting taking the supplement daily and 13% several times per week, according to AGES questionnaire. However, intake of milk or dairy products at baseline was not associated with vitamin D status in our study. Notably, milk or dairy products were not fortified with vitamin D during the study period.

We measured volumetric QCT-BMD rather than areal BMD, but similar associations have been shown between fractures and BMD using these two measures [35], [36]. Similar to many previous studies [3], [12] we found a slight but significant positive trend between BMD in femoral neck and serum concentrations of 25(OH)D in both sexes. Again, only levels below 30 nmol/L were consistently significantly different from the referent, which is in concordance with our results regarding increases in hip fracture risk below 30 nmol/L. However, according to earlier studies the observed difference of 0.10-0.16 BMD z-scores might be expected to be associated with only modest increases in fracture risk [37], [38]. The two-fold increase in risk associated with low 25(OH)D in our study supports the contention that the relationship between vitamin D and fracture risk may indeed be non-linear [1]. It has been suggested that vitamin D may reduce fracture rates through additional mechanisms, independent of bone density [39]. Specifically, vitamin D supplements may effectively decrease the risk of falling, especially in the institutionally elderly, possibly through actions involving balance and muscle strength [20], [40].

It is well known that elderly men have higher mean BMD and lower risk for hip fractures than women [14], [27], as confirmed in our study. However, the risk increase associated with low 25(OH)D concentrations was comparable in men and women suggesting that vitamin D status is no less important for lowering fracture risk among elderly men than women.

Concerns have been raised over assays for 25(OH)D giving varying results, casting doubt on the comparability of specific values for vitamin D status [41]. Our study employed a common assay method for 25(OH)D, widely used in clinical settings. Thus we believe that our results are clinically relevant, even though the levels measured by the CLIA method may be systematically lower than those measured by HPLC. Furthermore, systematic errors in absolute serum levels should only affect interpretations of our findings in terms of absolute cut-off values but should not affect the magnitude and strength of the observed association.

The length of follow-up may also be a concern, as single baseline vitamin D values may not represent status many years later [42]. Indeed, the study by Looker [13] showed that vitamin D concentrations predicted major osteoporotic fracture risk only for 10 years of follow up. Importantly, our mean follow-up time was 5.4 years, with mean time to event 3.4 years. Frail individuals may also be selectively advised to take vitamin D supplements, diminishing observed risk. However, results from our 25(OH)D analysis were not available until at the end of follow up, and thus our subjects could not be informed of their status earlier.

The main strength of our study is the large number of elderly people, displaying a wide variation in serum 25(OH)D values, and sustaining a total of 261 hip fractures. Thus, robust associations could be obtained between vitamin D status and fracture risk. The main limitation to our study, however, is the single baseline measurement of 25(OH)D. Also, as this is an observational study, some residual confounding cannot be excluded. However, as hazard ratios for hip fracture were quite similar in unadjusted and fully adjusted data in both sexes, residual confounding is not likely to substantially affect our main results.

To conclude: Our results lend support to the overarching importance of maintaining serum 25(OH)D above 30 nmol/L for bone health of elderly people while potential benefits of having much higher levels could not be detected.
